# Sensor-Derived Glycemic Metrics in Women with Type 1 Diabetes Using Insulin Degludec Versus Other Basal Insulin Analogs During Pregnancy—A Post Hoc Analysis of the CopenFast Trial

**DOI:** 10.1177/26884844251378015

**Published:** 2025-09-15

**Authors:** Julie C. Søholm, Sidse K. Nørgaard, Kirsten Nørgaard, Tine D. Clausen, Peter Damm, Elisabeth R. Mathiesen, Lene Ringholm

**Affiliations:** ^1^ Center for Pregnant Women with Diabetes, Rigshospitalet, Copenhagen, Denmark.; ^2^ Department of Nephrology and Endocrinology, Rigshospitalet, Copenhagen, Denmark.; ^3^ Department of Clinical Medicine, Faculty of Health and Medical Sciences, University of Copenhagen, Copenhagen, Denmark.; ^4^ Novo Nordisk A/S, Søborg, Denmark.; ^5^ Steno Diabetes Center Copenhagen, Herlev, Denmark.; ^6^ Department of Gynecology, Fertility and Obstetrics, Rigshospitalet, Copenhagen, Denmark.

**Keywords:** pregnancy, type 1 diabetes, continuous glucose monitoring, basal insulin analog, hypoglycemia

## Abstract

**Aim:**

To explore sensor-derived glycemic metrics during pregnancy in women with type 1 diabetes using insulin degludec or other basal insulin analogs.

**Methods:**

A post hoc analysis of 87 pregnant women with type 1 diabetes using intermittently scanned continuous glucose monitoring and multiple daily injections, including basal insulin analogs in the CopenFast trial. Glycemic metrics, including mean sensor glucose, time in range (TIRp, 3.5–7.8 mmol/L), and time below range in pregnancy (TBRp, <3.5 mmol/L), were assessed from periconception to 37 completed weeks.

**Results:**

In total, 58 women used degludec and 29 women used other basal insulin analogs. At baseline (median = 9.5 gestational weeks, interquartile range = 9.0–11.0), hemoglobin A1c was 50 ± 9 versus 46 ± 6 (*p* = 0.04) mmol/mol, and diabetes duration was 16 (10–21) versus 9 (4–19) years (*p* = 0.35). The use of faster-acting insulin aspart and insulin aspart was equally distributed in the two groups. Glycemic metrics were comparable throughout pregnancy for 24 hours in both groups. During nighttime, mean sensor glucose was higher and TIRp was lower in women using degludec compared with women using other basal insulin analogs, while TBRp was above treatment targets in both groups throughout pregnancy. Severe hypoglycemia occurred in 1 (2%) versus 5 (17%) (*p* = 0.01). Birthweight standard deviation score was 1.5 ± 1.2 versus 0.8 ± 1.1 (*p* = 0.01).

**Conclusions:**

In this post hoc analysis, pregnant women with type 1 diabetes using degludec achieved lower nighttime TIRp, experienced less severe hypoglycemia, and delivered infants who were heavier and thereby had less appropriate size compared with women using other basal insulin analogs.

## Introduction

Insulin analogs are widely used as part of modern insulin therapy during pregnancy with the aim of obtaining good glycemic control with near-normal glucose levels to prevent pregnancy-related complications.^1–3^ Despite this, women with type 1 diabetes often spend more time above the recommended glycemic treatment targets in the majority of pregnancy, thus increasing the risk of pregnancy complications such as preterm delivery and fetal overgrowth.^4–8^

The ultra-long-acting insulin analog degludec (insulin degludec) is characterized by an improved pharmacological time–action profile compared with insulin glargine, resulting in a half-life of approximately 25 hours.^9–11^ Outside of pregnancy, the consistent glucose-lowering effect and the duration of action of >42 hours provide greater reduction in fasting plasma glucose levels with lower prevalence of nocturnal hypoglycemia compared with insulin glargine.^12^

The use of insulin degludec during pregnancy in women with type 1 diabetes has been evaluated in a randomized controlled trial (RCT)^13^ and in observational studies,^14,15^ showing similar maternal and neonatal outcomes as compared with other basal insulin analogs. At our center, women with type 1 diabetes who have obtained good glycemic control and who are satisfied with using insulin degludec before conception can continue to use this insulin during pregnancy.^14,15^ However, no studies have investigated how the use of insulin degludec influences continuously measured sensor-derived glycemic metrics in pregnant women with type 1 diabetes.

The CopenFast trial, an RCT evaluating the effect of faster-acting insulin aspart compared with insulin aspart during pregnancy in 203 women with type 1 or type 2 diabetes, demonstrated that women randomized to faster-acting insulin aspart achieved similar fetal growth and maternal hemoglobin A1c (HbA1c) levels with less severe hypoglycemia compared with women randomized to insulin aspart.^16^ As part of usual care trial, women continued their usual basal insulin, either insulin degludec or other basal insulin analogs, and a large part of the women with type 1 diabetes used intermittently scanned continuous glucose monitoring (isCGM) during the trial.^4,16,17^

Achieving glycemic metric targets in early pregnancy has been associated with lower risk of delivering large for gestational age (LGA) infants,^4,8^ and obtaining glycemic metric targets may be more important for predicting fetal overgrowth and other poor pregnancy outcomes than obtaining HbA1c target levels.^4^

We hypothesized that use of insulin degludec leads to more favorable glycemic metrics during nighttime with less hypoglycemia compared with other basal insulin analogs during pregnancy. Therefore, we aimed to explore sensor-derived glycemic metrics from periconception to 37 completed weeks in women with type 1 diabetes using insulin degludec versus other basal insulin analogs.

## Material and Methods

### Study design and population

This was a post hoc analysis of sensor-derived glycemic metrics based on prospectively collected data in women with type 1 diabetes using isCGM and multiple daily injections with basal insulin analogs initiated before conception and continued during pregnancy in the CopenFast trial. Women were included in the CopenFast trial between November 11, 2019, and May 10, 2022. The cohort has previously been described in detail.^16,18^

In the CopenFast trial,^16^ 150 women with type 1 diabetes used either isCGM (*n* = 124) or real-time continuous glucose monitoring (rtCGM) (*n* = 26). Data from isCGM were collected from periconception (defined as first day of last menstrual cycle) until delivery, while data from rtCGM were primarily collected at trial visits only. Hence, rtCGM data were excluded from the current analysis. At randomization at median 9.5 (interquartile range [IQR] = 9.0–11.0) gestational weeks, 122 women with type 1 diabetes used an isCGM initiated before pregnancy and 2 women initiated isCGM in early pregnancy. Of the 124 women using isCGM, 5 women with early fetal loss, 6 women without available isCGM data, and 26 women not treated with basal insulin analogs were excluded, leaving a total of 87 women with type 1 diabetes using basal insulin analogs for this post hoc analysis.

The majority (93%) used FreeStyle Libre without hypoglycemic alerts, while the remaining six women used FreeStyle Libre 2 with hypoglycemic alerts initiated before pregnancy. Seven women changed from Freestyle Libre to FreeStyle Libre 2 during pregnancy.

### Sensor-derived glycemic metrics

All isCGM data were uploaded to LibreView (Abbott Diabetes Care, Alameda, California, USA). The raw isCGM data spreadsheets, reporting glucose data every 15 minutes, were downloaded from first day of last menstrual cycle or the day of isCGM application in early pregnancy and until 37 completed gestational weeks (37 weeks + 0 days) for each woman. In case of preterm delivery (before 37 completed weeks), data were downloaded until delivery. The following glycemic metrics were calculated: mean sensor glucose, mean sensor glucose coefficient of variation (CV), time in range in pregnancy (TIRp 3.5–7.8 mmol/L), time above range in pregnancy (TARp >7.8 mmol/L), and time below range in pregnancy (TBRp <3.5 mmol/L). Mean sensor glucose CV (%) was standard deviation (SD) divided by the mean of the corresponding glucose reading and multiplied by 100.

### Routine diabetes and pregnancy care

All women followed the routine diabetes and antenatal care program for pregnant women with diabetes at our center. Briefly, the women were seen for clinical visits approximately every second week^16,18^ where insulin doses were titrated aiming for isCGM targets of 4.0–5.5 mmol/L preprandially and 4.0–7.0 mmol/L postprandially, as well as mean sensor glucose values between 5 and 6 mmol/L, TIRp >70%, TARp <25%, and TBRp <4%.^16,18,19^ HbA1c targets were <48 mmol/mol (6.5%) before 20 weeks and <38 mmol/mol (5.6%) after 20 weeks.^16^ Women were recommended to evaluate sensor-derived glycemic metrics to adjust insulin dose every 3–5 days between routine visits to obtain the recommended isCGM targets. All women received the same recommendations on medical nutritional therapy, carbohydrate counting, and physical activity.^16,18,20^ Insulin degludec or other basal insulin analogs were administered once daily, but if indicated, the dose of other basal insulin analogs was given twice daily (*i.e.*, in the morning and in the evening) to obtain the recommended isCGM targets. Women were recommended to aim for gestational weight gain as follows: 10–15 kg if prepregnancy body mass index (BMI) <25 kg/m^2^, 5–8 kg if prepregnancy BMI 25–29.9 kg/m^2^, and 0–5 kg if prepregnancy BMI ≥30 kg/m^2^.^20^ Gestational weight gain was calculated from the last weight measured, often at 35 weeks, and self-reported prepregnancy weight.^21^ The women attended routine obstetric visits at approximately 10, 12, 21, 27, 33, and 36 weeks. Fetal growth was assessed by ultrasound at 27, 33, and 36 weeks, with more frequent assessments if indicated.

### Data collection and definitions

At the trial visits in the CopenFast trial at approximately 9, 21, 33, and 35 weeks, data on gestational age, HbA1c, body weight, blood pressure, insulin dose, presence of albuminuria, number of self-reported events of mild hypoglycemia (events with symptoms familiar to the woman as hypoglycemia and managed by herself^22^) the previous week, and events of severe hypoglycemia (requiring third party assistance^23^) since last trial visit were recorded. If a woman experienced severe hypoglycemia from randomization until delivery, a structured interview about the event was conducted.^16,18^ Diabetic retinopathy was assessed by retinal photo screening in early pregnancy and, when indicated, later in pregnancy.^24^

Excessive gestational weight gain was defined as exceeding the recommendations from the National Academy of Medicine (prepregnancy BMI <25 kg/m^2^: ≥16 kg, prepregnancy BMI 25–29.9 kg/m^2^: ≥11.5 kg, and prepregnancy BMI ≥30 kg/m^2^: >9.0 kg).^3^

Presence of preeclampsia (office blood pressure ≥140/90 mmHg with presence of albuminuria or new onset of symptoms or other signs of maternal organ dysfunction after 20 weeks),^25^ gestational hypertension (hypertension after 20 weeks without fulfilling the criteria for preeclampsia),^25^ mode of delivery, gestational age at delivery given as weeks (+days), birthweight, offspring sex, admission to the neonatal intensive care unit, and neonatal hypoglycemia (plasma glucose <2.2 mmol/L 2 hours after birth) were recorded. Birth weight SD score was calculated to adjust for gestational age and sex based on growth curves usually used in Scandinavia.^26^ LGA and small for gestational age were defined as birthweight >90th percentile and <10th percentile, respectively.^26^

### Statistical analysis

Continuous data with normal distribution were presented as mean (SD), and continuous data with skewed distribution were presented as median (IQR). Continuous skewed data were log-transformed, and after transformation, all variables, except duration of diabetes, satisfied normality.

Continuous outcomes were analyzed by linear regression analysis. The assumptions for linear regression were addressed by quantile–quantile plots and histogram of residuals. Categorical variables including number of women with severe hypoglycemia were analyzed using logistic regression analysis. The number of severe hypoglycemic events was presented as summarized counts.

The mean sensor-derived glycemic metrics, mean sensor glucose, mean sensor glucose CV, TIRp, TARp, and TBRp, were calculated for each woman and, based on trial visits in the CopenFast trial, where routine fetal ultrasound scans took place,^16^ analyzed in the following pregnancy intervals: from periconception to 9 completed weeks, from 10 to 21 completed weeks, from 22 to 33 completed weeks, and from 34 to 37 completed weeks. The sensor-derived glycemic metrics data were compared between women using insulin degludec and other basal insulin analogs for 24 hours, daytime (06:00 to 23:59) and nighttime (00:00 to 05:59).^27^ Weekly 24 hours glycemic summary metrics were calculated for each woman and for each gestational week from periconception until 37 completed weeks and plotted for women using insulin degludec versus other basal insulin analogs.

Correction for multiple testing was not performed.

A two-sided *p*-value <0.05 was regarded as statistically significant.

The statistical analyses were made using R version 4.1.0 (R Core Team, 2021; R Foundation for Statistical Computing, Vienna, Austria).^28^

### Ethics

Written consent was obtained from all women, and the trial protocol was approved by the Danish Medicines Agency (2018–004680-31) and the Regional Ethics Committee (H-19029966).

This trial is registered with ClinicalTrials.gov, NCT03770767.

## Results

In total, 58 (67%) women used insulin degludec once daily and 29 (33%) women used other basal insulin analogs once or twice daily (insulin glargine [*n* = 26] or insulin detemir [*n* = 3]) initiated before pregnancy and used until delivery. At baseline, HbA1c was higher (50 ± 9 vs. 46 ± 6 mmol/mol; 6.7 ± 0.7 vs. 6.4 ± 0.3%; *p* = 0.04), while allocation to trial medication faster-acting insulin aspart or insulin aspart was similar between women using insulin degludec or other basal insulin analogs, respectively (Table 1).

**Table 1.** Baseline Characteristics in 87 Women with Type 1 Diabetes Using Intermittently Scanned Continuous Glucose Monitoring and Insulin Degludec or Other Basal Insulin Analogs from Periconception to 37 Completed Weeks

	Insulin degludec (*n* = 58)	Other basal insulin analogs (*n* = 29)	*p*-Value
Age, years	31.5 ± 5	32.5 ± 6	0.35
Duration of diabetes, years	16.0 (10.0–21.0)	10.0 (4.5–19.0)	0.09
HbA1c, mmol/mol	50 ± 9	46 ± 6	0.04
HbA1c, %	6.7 ± 0.6	6.4 ± 0.3	
Prepregnancy weight, kg	68.0 (63.0–78.0)	68.0 (59.0–81.0)	0.91
Prepregnancy BMI, kg/m^2^	24.8 (21.9–27.4)	23.8 (21.4–28.0)	0.73
Normal (<25 kg/m^2^)	30 (52)	17 (58)	
Overweight (25–30 kg/m^2^)	21 (36)	8 (28)	
Obese (>30 kg/m^2^)	7 (12)	4 (14)	
Nulliparous	29 (50)	11 (38)	0.40
Nordic origin	55 (95)	25 (86)	0.22
Allocation to trial medication			0.93
Faster-acting insulin aspart	32 (55)	15 (52)	
Insulin aspart	26 (45)	14 (48)	
Number of women with severe hypoglycemia in the year preceding pregnancy	1 (2)	1 (4)	1.00
Normal hypoglycemia awareness in early pregnancy^a^	25 (57)	10 (48)	0.67
Systolic office blood pressure, mmHg	113 ± 10	111 ± 11	0.27
Diastolic office blood pressure, mmHg	74 ± 7	72 ± 6	0.08
Diabetic retinopathy	17 (29)	9 (31)	0.99
Albuminuria, albumin/creatinine ratio >30 mg/g	3 (5)	0 (0)	0.55

Data are presented as median (interquartile range), mean (standard deviation), or *n* (%).

^a^ Data on hypoglycemia awareness were obtained from a questionnaire where 62 women (72%) responded. Self-estimated hypoglycemia awareness was defined as normal, when the woman answered “always” to the question: “Do you recognize symptoms, when you have a hypoglycemic event?”

BMI, body mass index; HbA1c, hemoglobin A1c.

The dataset consisted of 2,214,347 glucose measurements over 24 hours and 485,541 measurements during nighttime.

**FIG. 1.**
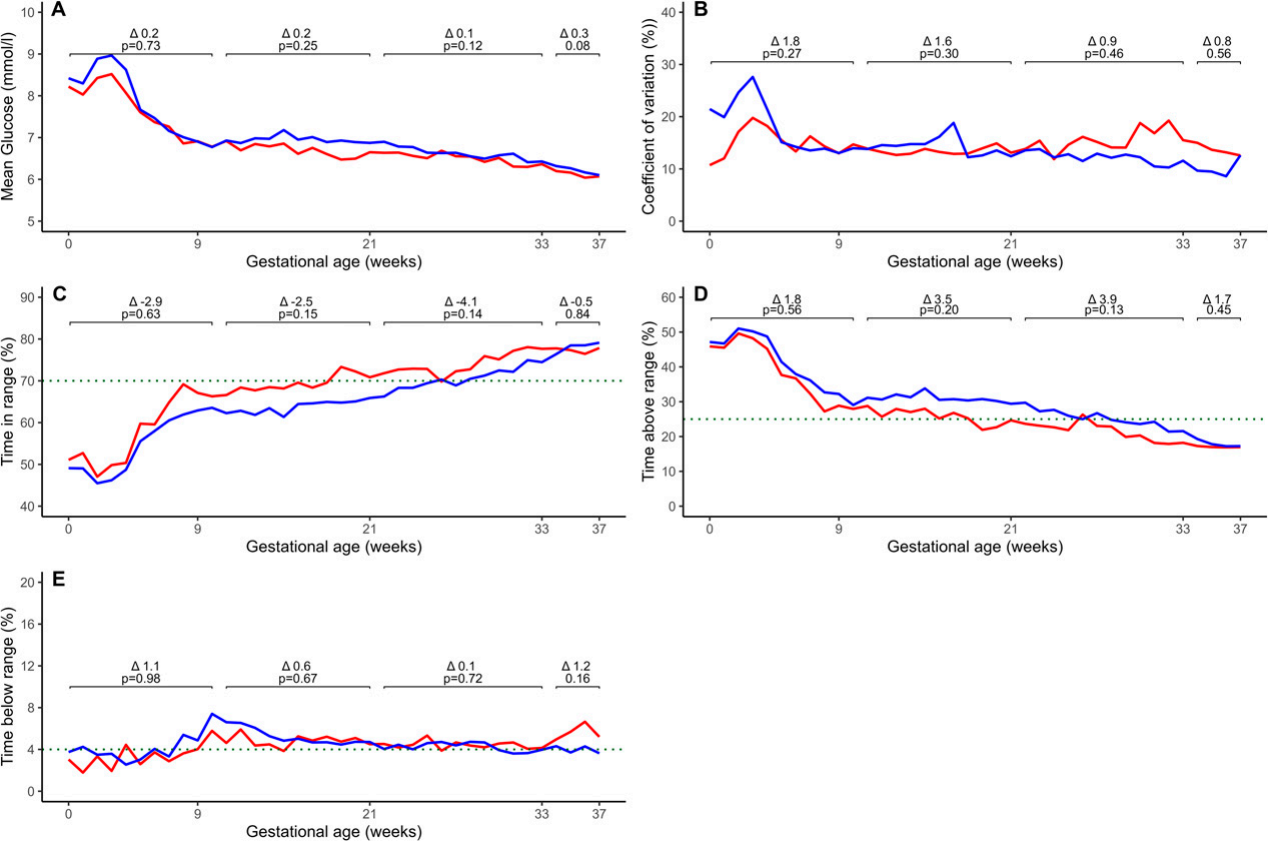
Sensor-derived glycemic metrics from periconception to 37 completed weeks in 87 women with type 1 diabetes using intermittently scanned continuous glucose monitoring according to insulin treatment with insulin degludec (blue) or other basal insulin analogs (red). The mean sensor-derived glycemic metrics were calculated for each woman for each gestational week and plotted according to insulin treatment with insulin degludec or other basal insulin analogs. Data on sensor-derived glycemic metrics presented in the brackets were calculated as the mean value of each woman’s average and were analyzed in pregnancy intervals: from conception to 9 completed weeks, from 10 to 21 completed weeks, from 22 to 33 completed weeks, and from 34 to 37 completed weeks and compared between the two groups. Data were available for 86% (*n* = 75), 100% (*n* = 87), 100% (*n* = 87), and 94% (*n* = 82) in the pregnancy intervals. **(A)** Mean sensor glucose (mmol/L). **(B)** Mean glucose coefficient of variation (%). **(C)** Mean time in range in pregnancy (3.5–7.8 mmol/L) (%). **(D)** Mean time above range in pregnancy (>7.8 mmol/L) (%). **(E)** Mean time below range in pregnancy (<3.5 mmol/L) (%).

Over 24 hours and during daytime, mean sensor glucose, mean sensor CV, TIRp, and TARp improved throughout pregnancy, while TBRp remained stable and close to treatment targets in both groups. Sensor-derived glycemic metrics over 24 hours and during daytime were similar between the two groups (Fig. 1A–E, Table 2). TIRp >70% was achieved by both groups in the pregnancy interval from 22 to 33 completed weeks. After 24 hours, TARp <25% was achieved from 34 completed weeks in women using insulin degludec and from 22 to 33 completed weeks in women using other basal insulin analogs (Fig. 1D).

**Table 2.** Sensor-Derived Glycemic Metrics According to Daytime (06:00–23:59) and Nighttime (00:00–05:59) in 87 Women with Type 1 Diabetes Using Intermittently Scanned Continuous Glucose Monitoring and Insulin Degludec or Other Basal Insulin Analogs from Periconception to 37 Completed Weeks

	Daytime (06:00–23:59)					Nighttime (00:00–05:59)				
Glycemic metrics	Insulin degludec (*n* = 58)	Other basal insulin analogs (*n* = 29)	Estimated mean difference (95% CI)	*p*-Value	Insulin degludec (*n* = 58)	Other basal insulin analogs (*n* = 29)	Estimated mean difference (95% CI)	*p*-Value		
Periconception to 9 completed weeks								
Mean sensor glucose, mmol/L	7.5 ± 1.1	7.6 ± 1.2	−0.1 (−0.6, 0.6)	0.88	7.1 ± 1.5	6.5 ± 1.2	0.6 (−0.9, 1.4)	0.08
TIRp 3.5–7.8 mmol/L (63–140 mg/dL), %	57.4 ± 14.1	59.8 ± 17.2	−2.4 (−9.7, 5.2)	0.66	58.9 ± 17.6	67.5 ± 16.5	−8.6 (−18.1, 0.8)	0.09
TARp >7.8 mmol/L (>140 mg/dL), %	37.9 ± 17.5	37.1 ± 17.5	0.8 (−7.2, 8.8)	0.84	33.9 ± 19.5	25.2 ± 16.1	8.5 (−0.9, 18.4)	0.10
TBRp <3.5 mmol/L (<64 mg/dL), %	4.5 ± 4.2	3.1 ± 3.6	1.4 (−0.6, 3.4)	0.14	7.2 ± 7.8	7.3 ± 8.5	−0.1 (−4.8, 4.6)	0.98
From 10 to 21 completed weeks								
Mean sensor glucose, mmol/L	7.1 ± 0.9	7.0 ± 0.8	0.1 (−0.3, 0.5)	0.82	6.7 ± 1.0	6.0 ± 0.8	0.7 (0.4, 1.2)	0.002
TIRp 3.5–7.8 mmol/L (63–140 mg/dL), %	63.3 ± 13.1	66.6 ± 15.2	−3.3 (−9.7, 2.9)	0.42	63.0 ± 16.8	74.0 ± 13.4	−11.0 (−18.1, −3.9)	0.003
TARp >7.8 mmol/L (>140 mg/dL), %	32.7 ± 13.5	30.8 ± 14.4	1.9 (−3.8, 8.8)	0.54	28.8 ± 15.8	17.1 ± 11.2	11.7 (5.3, 18.2)	0.004
TBRp <3.5 mmol/L (<64 mg/dL), %	4.0 ± 3.1	3.3 ± 2.6	0.7 (−0.5, 2.2)	0.24	8.2 ± 6.4	8.9 ± 7.1	−0.7 (−3.8, 2.4)	0.48
From 22 to 33 completed weeks								
Mean sensor glucose, mmol/L	6.7 ± 0.7	6.8 ± 0.7	0.1 (−0.2, 0.5)	0.88	6.2 ± 0.8	5.7 ± 0.6	0.6 (0.2, 0.9)	0.002
TIRp 3.5–7.8 mmol/L (63–140 mg/dL), %	69.4 ± 12.5	70.2 ± 13.5	−0.8 (−8.2, 3.6)	0.81	72.6 ± 12.0	80.7 ± 12.6	−8.1 (−13.7, −2.6)	0.005
TARp >7.8 mmol/L (>140 mg/dL), %	27.1 ± 12.1	25.2 ± 14.6	1.9 (−3.8, 7.6)	0.50	20.8 ± 11.7	12.2 ± 9.4	8.6 (3.7, 13.6)	<0.001
TBRp <3.5 mmol/L (<64 mg/dL), %	3.6 ± 2.8	3.1 ± 1.9	0.5 (−0.8, 1.6)	0.49	6.6 ± 5.5	7.1 ± 5.8	−0.5 (−3.2, 2.2)	0.91
From 34 to 37 completed weeks								
Mean sensor glucose, mmol/L	6.4 ± 0.5	6.3 ± 0.7	0.1 (−0.2, 0.4)	0.98	5.9 ± 0.7	5.4 ± 0.6	0.5 (0.2, 0.8)	0.01
TIRp 3.5–7.8 mmol/L (63–140 mg/dL), %	76.1 ± 10.1	73.4 ± 14.4	2.7 (−4.6, 6.2)	0.39	80.2 ± 10.5	82.4 ± 12.2	−2.2 (−7.3, 3.2)	0.44
TARp >7.8 mmol/L (>140 mg/dL), %	21.4 ± 9.7	21.4 ± 14.6	1.0 (−5.1, 5.2)	0.53	13.3 ± 10.0	8.6 ± 6.7	4.8 (3.6, 13.6)	0.03
TBRp <3.5, mmol/L (<64 mg/dL), %	3.6 ± 3.1	4.3 ± 4.2	−0.7 (−2.6, 0.9)	0.32	6.5 ± 7.2	9.0 ± 8.8	−2.5 (−6.4, 0.8)	0.13

Results are given as mean ± standard deviation. Data were available for 86% (*n* = 75), 100% (*n* = 87), 100% (*n* = 87), and 94% (*n* = 82) in the pregnancy intervals from periconception to 9 completed weeks, from 10 to 21 completed weeks, from 22 to 33 completed weeks, and from 34 to 37 completed weeks. CI, confidence interval; TARp, time above range in pregnancy; TBRp, time below range in pregnancy; TIRp, time in range in pregnancy.

During nighttime, all sensor-derived glycemic metrics were similar between the groups from periconception until 9 completed weeks. From 10 to 37 completed weeks, women using insulin degludec had 0.5–0.7 mmol/L higher mean sensor glucose, 4.8%–11.7% higher TARp, and 2.2%–11.0% lower TIRp (estimated treatment differences). TBRp was similar and higher than treatment target throughout pregnancy in both groups (Table 2).

Both HbA1c levels at 35 weeks (Table 3) and the number of mild hypoglycemic events (Table 4) were similar throughout pregnancy. The number of women experiencing ≥1 severe hypoglycemic event from periconception to 37 completed weeks was 1 (2%) versus 5 (17%) (*p* = 0.01) (Table 3). The total number of severe hypoglycemic events from periconception to 37 completed weeks was 1 versus 9. From randomization to delivery, 60% of severe hypoglycemic events occurred during sleep.

**Table 3.** Maternal, Pregnancy, and Neonatal Outcomes in 87 Women with Type 1 Diabetes Using Intermittently Scanned Continuous Glucose Monitoring and Insulin Degludec or Other Basal Insulin Analogs from Periconception to Delivery

	Insulin degludec (*n* = 58)	Other basal insulin analogs (*n* = 29)	Estimated mean difference (95% CI) or odds ratio (95% CI)	*p*-Value
Maternal and pregnancy outcome				
HbA1c at 35 weeks, mmol/mol	45 ± 5	43 ± 6	2.0 (0.01;0.1)	0.10
HbA1c at 35 weeks, %	6.3 ± 0.2	6.1 ± 0.3		
Normal hypoglycemia awareness at 33 weeks^a^	22 (56)	6 (38)	1.2 (0.9, 1.6)	0.33
Number of women with severe hypoglycemia from periconception to delivery	1 (2)	5 (17)	0.85 (0.7, 0.9)	0.01
Preeclampsia	6 (10)	3 (7)	1.5 (0.3, 10.9)	0.65
Gestational weight gain, kg	14.0 ± 6	13.0 ± 7	1.0 (−2.0, 4.0)	0.55
Excessive gestational weight gain^b^	26 (45)	11 (38)	1.4 (0.6, 3.7)	0.50
Gestational age at birth, days	265 (260–269)	266 (260–270)	−1.0 (−4.7, 1.9)	0.49
Preterm delivery before 34 weeks	0 (0)	0 (0)	—	—
Preterm delivery before 37 weeks	9 (15)	4 (14)	1.4 (0.4, 6.6)	0.99
Neonatal outcome				
Birth weight, g	3721 ± 478	3490 ± 479	231 (11.7, 450.7)	0.04
Birth weight standard deviation score	1.5 ± 1.2	0.8 ± 1.1	0.7 (0.1, 1.3)	0.01
Large for gestational age	33 (57)	11 (38)	2.1 (0.8, 5.5)	0.15
Small for gestational age	0 (0)	0 (0)	—	—
Admission to neonatal care unit	15 (26)	3 (10)	2.7 (0.8, 12.6)	0.23
Neonatal glucose levels 2 hours after delivery, mmol/L	3.5 ± 1.1	3.1 ± 1.1	−0.4 (−0.7, 0.2)	0.08

Data are presented as median (interquartile range), mean (standard deviation), or *n* (%). Data were available >95% of the women unless otherwise stated.

^a^ Data on hypoglycemia awareness were obtained from a questionnaire where 55 women (63%) responded. Self-estimated hypoglycemia awareness was defined as normal, when the woman answered “always” to the question: “Do you recognize symptoms, when you have a hypoglycemic event?”

^b^ Excessive gestational weight gain: Exceeding the recommended gestational weight gain according to prepregnancy BMI recommended by the National Academy of Medicine (prepregnancy BMI <25 kg/m^2^: ≥16 kg, prepregnancy BMI 25–29.9 kg/m^2^: ≥11.5 kg, and prepregnancy BMI ≥30 kg/m^2^: >9.0 kg).

**Table 4.** Insulin Dose and Mild Hypoglycemia in Women with Type 1 Diabetes Using Intermittently Scanned Continuous Glucose Monitoring and Insulin Degludec or Other Long-Acting Insulin Analogs from Periconception to 37 Completed Weeks

	Insulin degludec (*n* = 58)	Other basal insulin analogs (*n* = 29)	*p*-Value
Before pregnancy			
Basal insulin dose			
IU/24 hours	22.4 ± 9.8	21.8 ± 8.4	0.68
IU/kg/24 hours	0.31 ± 0.13	0.30 ± 0.11	0.69
Meal-time insulin dose			
IU/24 hours	16.8 ± 8.6	18.4 ± 10.8	0.60
IU/kg/24 hours	0.24 ± 0.10	0.25 ± 0.12	0.64
Total daily insulin dose			
IU/24 hours	39.2 ± 15.3	40.2 ± 15.9	0.93
IU/kg/24 hours	0.56 ± 0.20	0.55 ± 0.18	0.85
At randomization (9 weeks)			
Basal insulin dose			
IU/24 hours	19.4 ± 9.1	18.5 ± 8.4	0.64
IU/kg/24 hours	0.26 ± 0.09	0.24 ± 0.10	0.46
Meal-time insulin dose			
IU/24 hours	17.8 ± 10.1	17.3 ± 8.1	0.91
IU/kg/24 hours	0.24 ± 0.08	0.23 ± 0.1	0.94
Total daily insulin dose			
IU/24 hours	37.2 ± 17.5	35.8 ± 17.0	0.95
IU/kg/24 hours	0.50 ± 0.14	0.48 ± 0.15	0.86
Events of mild hypoglycemia in the previous week	5 (3–9)	6 (3.5–9.5)	0.75
At 21 weeks			
Basal insulin dose			
IU/24 hours	20.0 ± 11.5	20.2 ± 10.5	0.94
IU/kg/24 hours	0.25 ± 0.11	0.26 ± 0.12	0.82
Meal-time insulin dose			
IU/24 hours	24.1 ± 13.2	23.2 ± 13.0	0.56
IU/kg/24 hours	0.31 ± 0.15	0.30 ± 0.14	0.82
Total daily insulin dose			
IU/24 hours	44.1 ± 19.7	43.4 ± 19.7	0.83
IU/kg/24 hours	0.54 ± 0.21	0.56 ± 0.22	0.76
Event of mild hypoglycemia in the previous week	4 (3–7)	5 (2–7)	0.89
At 33 weeks			
Basal insulin dose			
IU/24 hours	28.0 ± 16.4	31.2 ± 20.2	0.44
IU/kg/24 hours	0.32 ± 0.16	0.39 ± 0.23	0.22
Meal-time insulin dose			
IU/24 hours	41.8 ± 20.4	46.5 ± 23.2	0.32
IU/kg/24 hours	0.49 ± 0.21	0.57 ± 0.23	0.11
Total daily insulin dose			
IU/24 hours	69.8 ± 32.2	77.7 ± 35.8	0.45
IU/kg/24 hours	0.82 ± 0.31	0.97 ± 0.37	0.08
Events of mild hypoglycemia in the previous week	3.5 (2–5)	4 (2–5)	0.48
At 35 weeks^a^			
Basal insulin dose			
IU/24 hours	25.4 ± 17.0	32.4 ± 23.9	0.08
IU/kg/24 hours	0.29 ± 0.15	0.37 ± 0.27	0.12
Meal-time insulin dose			
IU/24 hours	42.0 ± 22.1	48.3 ± 23.9	0.38
IU/kg/24 hours	0.49 ± 0.23	0.56 ± 0.29	0.15
Total daily insulin dose			
IU/24 hours	67.4 ± 34.5	80.7 ± 35.5	0.07
IU/kg/24 hours	0.77 ± 0.32	0.93 ± 0.41	0.08
Events of mild hypoglycemia in the previous week	3 (2–5)	3 (1–7)	0.61

Data are presented as median (interquartile range) or mean (standard deviation). Data were available >95% of the women unless otherwise stated.

^a^ Data were available for 92% (*n* = 80) of women using insulin degludec or other basal insulin analogs.

Insulin doses were similar throughout pregnancy between the groups (Table 4).

Infant birthweight was 3721 ± 478 versus 3490 ± 479 g, *p* = 0.04, and birthweight SD score was 1.5 ± 1.2 versus 0.8 ± 1.1 (*p* = 0.01). All other pregnancy and neonatal outcomes were similar between the groups (Table 3).

## Discussion

This post hoc analysis of the CopenFast trial included 87 women with type 1 diabetes who used multiple daily injections and continuous isCGM from periconception to 37 completed weeks. Women using insulin degludec exhibited similar 24 hours glycemic metrics and higher nocturnal glucose levels compared with those using other basal insulin analogs. Additionally, women using insulin degludec delivered infants who were heavier and thereby had less appropriate size compared with women using other basal insulin analogs who, in contrast, experienced more severe hypoglycemic events.

This analysis offers novel insight into sensor-derived glycemic metrics and pregnancy outcomes in women with type 1 diabetes who continue using their usual basal insulin analogs during pregnancy.

Overall, sensor-derived glycemic metrics improved similarly over 24 hours and during daytime from periconception to 37 completed weeks in both groups, reflecting glucose levels closer to targets. However, during nighttime, women using insulin degludec had higher glucose levels throughout most of the pregnancy, with a similar percentage of TBRp compared with those using other basal insulin analogs. The relatively high percentage of nighttime TBRp in both groups might limit the increase in basal insulin doses, thereby influencing other sensor-derived glycemic metrics both over 24 hours and at nighttime.

Achieving glycemic metric targets early in pregnancy is associated with a lower risk of delivering LGA infants.^4,8^ However, neither group achieved a mean sensor glucose at target between 5 and 6 mmol/L during pregnancy, and TIRp >70% was not reached until the pregnancy interval 22–33 weeks.

The seemingly disappointing effect of insulin degludec on sensor-derived glycemic metrics during nighttime may be ascribed to confounding by indication, as the women treated with insulin degludec before conception may previously have been using other basal insulin analogs and having challenging glycemic control, which were indications for switching to insulin degludec prior to the CopenFast trial. In contrast, the women using other basal insulin analogs were characterized by well-controlled diabetes before pregnancy and a rather short duration of diabetes, suggesting that they *a priori* had a better glycemic control since they did not meet the criteria for switching to insulin degludec or insulin pump therapy before pregnancy.

Pregnancy in women with type 1 diabetes is characterized by alterations in insulin requirements from week to week. As expected, insulin doses increased gradually during pregnancy, which was mainly ascribed to increasing meal-time insulin doses and to a smaller extent basal insulin doses.^29–31^

Even though total insulin doses, and in particular basal insulin doses, were similar between groups throughout pregnancy, nocturnal TIRp was significantly lower from 10 to 33 completed weeks in women using insulin degludec compared with women using other basal insulin analogs. We acknowledge that data on corrective insulin doses for episodes with elevated glucose levels were not available. Insulin degludec was administered once daily according to the long duration of action and the manufacturer’s product information,^10^ while other basal insulin analogs with a shorter duration of action were administered once or twice daily based on individual judgment. It remains speculative whether once or twice daily administration of insulin degludec or other basal insulin analogs, respectively, affected the presented results.

The use of FreeStyle Libre without hypoglycemia alerts during pregnancy has been associated with higher nighttime TBRp compared with another type of continuous glucose monitoring (CGM) used in the same pregnant women with type 1 diabetes.^32^ This may explain the relatively high TBRp exceeding the target during nighttime observed in this analysis. Whether this also applies to the newer types of CGM (FreeStyle Libre 2 and FreeStyle Libre 3) remains to be investigated. Surprisingly, the prevalence of weekly mild hypoglycemia events was similar in both groups. Mild hypoglycemia during nighttime was not recorded.

As in the original trial,^16^ the number of women reporting severe hypoglycemia was low, especially among those using insulin degludec. Most severe hypoglycemic events occurred in women using other basal insulin analogs. During pregnancy in women with type 1 diabetes, severe hypoglycemia frequently occurs during sleep,^22^ and this was also the case in this study. The low incidence of severe hypoglycemia in women using insulin degludec is reassuring. However, it is unclear whether this is due to a protective effect of insulin degludec or because these women had higher HbA1c levels in early pregnancy. Despite this uncertainty, our data suggest that insulin degludec may be titrated similarly to other basal insulin analogs without increased risk of severe hypoglycemia.

Both birthweight and birthweight SD score were significantly higher in infants born to women using insulin degludec compared with infants born to women using other basal insulin analogs. In the EXPECT trial, a multicenter RCT in women with type 1 diabetes, treatment with insulin degludec, or insulin detemir in combination with insulin aspart resulted in similar glycemic control and pregnancy outcomes.^13^ Given that in the present analysis, women using insulin degludec were characterized by higher HbA1c before pregnancy and a numerically longer duration of diabetes, the groups were not perfectly matched, and larger studies using propensity score matching of the cohorts with the use of newer generations of CGM that are more precise are needed to confirm or reject the findings of this post hoc analysis.

Strengths of this study include the prospectively collected data from periconception to 37 completed weeks in women with type 1 diabetes using insulin degludec or other basal insulin analogs initiated before pregnancy. The cohort consisted of pregnant women with mainly good glycemic control who were treated according to the same treatment recommendations. isCGM data were continuously collected from periconception to 37 completed weeks, and large amounts of data were included in this analysis. Data on rtCGM were not collected continuously during pregnancy in the small subset of women using this device, and rtCGM data were therefore not included in this post hoc analysis.

Limitations include the cohort originating from a single center with specialized expertise in managing diabetes during pregnancy, despite being from a well-defined geographical area. This may restrict the generalizability of the results to other populations. Adjustment for multiple testing was not performed, and therefore, there is a risk of a type 1 statistical error, and the number of included women in the study is limited.

To conclude, in this post hoc analysis, pregnant women with type 1 diabetes using insulin degludec achieved lower TIRp during nighttime, experienced less severe hypoglycemia, and delivered infants who were heavier and thereby had less appropriate size compared with women using other basal insulin analogs. Further studies, including glycemic metrics separated in daytime and nighttime for evaluating the impact of long-acting insulin analogs during pregnancy, are warranted.

## Data Availability

The dataset analyzed during the study is available from the corresponding author upon reasonable request.

## Abbreviations Used

CV: coefficient of variation
HbA1c: hemoglobin A1c
isCGM: intermittently scanned continuous glucose monitoring
LGA: large for gestational age
RCT: randomized controlled trial
rtCGM: real-time continuous glucose monitoring
TARp: Time above range in pregnancy
TBRp: Time below range in pregnancy
TIRp: Time in range in pregnancy
